# The Effects of Virtual Reality Treatment on Prefrontal Cortex Activity in Patients With Social Anxiety Disorder: Participatory and Interactive Virtual Reality Treatment Study

**DOI:** 10.2196/31844

**Published:** 2021-12-17

**Authors:** Hojun Lee, JongKwan Choi, Dooyoung Jung, Ji-Won Hur, Chul-Hyun Cho

**Affiliations:** 1 Department of Psychiatry School of Medicine Keimyung University Daegu Republic of Korea; 2 Department of Psychiatry Keimyung University Dongsan Medical Center Daegu Republic of Korea; 3 OBELAB Inc Seoul Republic of Korea; 4 Department of Biomedical Engineering Ulsan National Institute of Science and Technology Ulsan Republic of Korea; 5 School of Psychology Korea University Seoul Republic of Korea; 6 Department of Psychiatry College of Medicine Chungnam National University Daejeon Republic of Korea; 7 Department of Psychiatry Chungnam National University Sejong Hospital Sejong Republic of Korea

**Keywords:** anxiety, social anxiety disorder, virtual reality, fNIRS, brain activity, prefrontal cortex, effectiveness

## Abstract

**Background:**

Attempts to use virtual reality (VR) as a treatment for various psychiatric disorders have been made recently, and many researchers have identified the effects of VR in psychiatric disorders. Studies have reported that VR therapy is effective in social anxiety disorder (SAD). However, there is no prior study on the neural correlates of VR therapy in patients with SAD.

**Objective:**

The aim of this study is to find the neural correlates of VR therapy by evaluating the treatment effectiveness of VR in patients with SAD using portable functional near-infrared spectroscopy (fNIRS).

**Methods:**

Patients with SAD (n=28) were provided with 6 sessions of VR treatment that was developed for exposure to social situations with a recording system of each participant’s self-introduction in VR. After each VR treatment session, the first-person view (video 1) and third-person view (video 2) clips of the participant’s self-introduction were automatically generated. The functional activities of prefrontal regions were measured by fNIRS while watching videos 1 and 2 with a cognitive task, before and after whole VR treatment sessions, and after the first session of VR treatment. We compared the data of fNIRS between patients with SAD and healthy controls (HCs; n=27).

**Results:**

We found that reduction in activities of the right frontopolar prefrontal cortex (FPPFC) in HCs was greater than in the SAD group at baseline (*t*=–2.01, *P*=.049). Comparing the frontal cortex activation before and after VR treatment sessions in the SAD group showed significant differences in activities of the FPPFC (right: *t*=–2.93, *P*<.001; left: *t*=–2.25, *P*=.03) and the orbitofrontal cortex (OFC) (right: *t*=–2.10, *P*=.045; left: *t*=–2.21, *P*=.04) while watching video 2.

**Conclusions:**

Activities of the FPPFC and OFC were associated with symptom reduction after VR treatment for SAD. Our study findings might provide a clue to understanding the mechanisms underlying VR treatment for SAD.

**Trial Registration:**

Clinical Research Information Service (CRIS) KCT0003854; https://tinyurl.com/559jp2kp

## Introduction

Social anxiety disorder (SAD) is a common psychiatric disease, with 8.4%-15% of the population worldwide diagnosed with it [1]. According to the *Diagnostic and Statistical Manual of Mental Disorders, Fifth Edition* (DSM-5), SAD is characterized by fear or anxiety in situations in which people receive negative attention from others [2]. Individuals with SAD avoid social situations and have difficulties in maintaining interpersonal relationships, with serious impairment in academic, occupational, and social functions [3]. Comorbidities, such as depressive disorders, anxiety disorders, substance use disorder, obsessive compulsive disorder, and avoidant personality disorder, typically accompany SAD. Moreover, patients are likely to be single and unemployed and develop suicidal ideas, low self-esteem and a low quality of life, and chronic illnesses without treatment [4,5].

Effective pharmacological agents for SAD treatment include selective serotonin reuptake inhibitors, serotonin and norepinephrine reuptake inhibitors, benzodiazepines, and beta-adrenergic antagonists. Furthermore, nonpharmacological therapies, such as cognitive behavior therapy (CBT) or social skill training, and a combination of pharmacological and nonpharmacological therapies are considered treatment modalities [6-8]. Although these treatments are effective, patients with SAD may be reluctant to receive psychiatric medication, have difficulty in visiting the treatment room, and drop out of treatment to avoid social situations or spatial constraints. Thus, patients may not actively participate in treatments [9,10].

To overcome the limitations of conventional therapeutics, digital intervention strategies, such as virtual reality (VR), that incorporate advanced technology into conventional therapies have been developed. Since the 1990s, VR has been used in various clinical treatments in medicine. However, initially, it could not reproduce reality well due to technical limitations, but with gradual developments in digital technologies, such as graphics and sounds, the treatment effects of realistic VR have improved considerably [11-13].

Since Rothbaum et al [14] reported the effects of progressive exposure therapy using VR in acrophobia, many studies have reported the effects of VR in various psychiatric disorders, such as specific phobia, posttraumatic stress disorder, SAD, attention deficit hyperactivity disorder, autism spectrum disorder, schizophrenia, depressive disorders, anxiety disorders, eating disorders, addiction, and mild cognitive impairment [13,15-19]. VR therapy has numerous advantages because it enables self-directed treatment at home or in locations where the patient feels comfortable. Furthermore, VR motivates patients toward treatment, mitigating the limitations of conventional therapist-led therapy [15,20,21]. VR can depict various social situations that cannot be reproduced in real treatment spaces because of spatial or human constraints and allows modification of patient treatment for specific situations [22].

Although VR treatment for SAD has attracted considerable attention, it is still a novel treatment modality. Pertaub et al [23] identified that social anxiety can be induced by virtual situations, which yielded results similar to conventional exposure therapy. Robilard et al [24] reported improved detection of anxiety symptoms with VR-CBT in patients with SAD. Randomized controlled trials have reported the effects of VR exposure therapy in patients with SAD [25-27]. However, limited clinical studies have been performed for investigating the effectiveness of VR treatment, and most studies have only identified effectiveness based on psychological assessments that relied on patients’ subjective expressions [28].

In this study, we designed an advanced therapeutic tool compared with existing VR treatments. During the sessions, the participants’ own voices were recorded, and scenes were constructed with the participants’ self-introduction in first person and third person to introduce participatory and interactive features that could be used therapeutically. The use of this VR program for measuring the psychological scale changes in patients with SAD has been reported earlier in a study [29]. In this study, we examined changes and responses in brain frontal region activities using functional near-infrared spectroscopy (fNIRS). fNIRS can be used to examine the functional activity in certain areas of the brain by measuring changes in the concentrations of oxygenated hemoglobin (HbO_2_) and deoxygenated hemoglobin (HbR) in brain tissue to assess neurological activation. fNIRS is safe, portable, and easy to use compared with neuroimaging tools such as functional magnetic resonance imaging (fMRI) [30]. Previous neuroimaging studies have reported changes in the medial prefrontal cortex (MPFC), ventrolateral prefrontal cortex (VLPFC), amygdala, anterior cingulate cortex (ACC), and posterior cingulate cortex (PCC) in SAD. The changes in the prefrontal cortex (PFC) were reported before and after VR therapy and during treatment [31].

The purpose of this study is to identify the neural correlates of symptom improvement after VR treatment in SAD based on the efficacy of the VR program reported previously [29]. We used VR-derived video clips with first- or third-person interviews to observe changes in brain activity. The neural correlates of symptom improvement after VR treatment in patients with SAD were studied by analyzing and assessing changes in neuronal activities using fNIRS compared with healthy controls (HCs).

## Methods

### Study Participants

In total, 40 patients with SAD and 34 HCs matched for age, sex, and handedness were enrolled in the study. After data quality verification with fNIRS, we included 28 patients (19 [68%] women) diagnosed with primary SAD and 27 HCs (13 [48%] women) in the study. The SAD and HC groups were recruited from universities through online advertisements on bulletin boards. The inclusion criteria for the SAD group were as follows:

- Korean-speaking men or women aged between 19 and 31 years
- Satisfy DSM-IV criteria for SAD according to the Mini-International Neuropsychiatric Interview (MINI; a psychiatrist conducted MINI to select the SAD and HC groups in this study) [32]
- Psychotropic medication–naive patients without psychiatric comorbidities (excluding depressive and panic disorders)
- Patients not undergoing psychotherapy
- Patients without a history of neurological disorders
- Patients without a history of psychotic symptoms upon VR exposure
- Patients who experience epilepsy and are not vulnerable to visual stimuli

The exclusion criteria were as follows:

- History of intellectual disability or organic brain damage
- Individuals who experience psychotic symptoms upon VR experiences
- People vulnerable to visual stimuli
- People who are unsuitable for participation in fNIRS research (eg, people who cannot sustain the fNIRS test because of discomfort or anxiety in wearing fNIRS equipment or experiences of side effects, such as headache)

The HC group did not have preexisting neurological or psychiatric conditions. The Korean Social Avoidance and Distress (K-SAD) scale was prepared using an online questionnaire, and patients with a score of 82 or above were enrolled in the SAD group. All participants provided informed written consent after explanation of the study procedures and were required to pass an fNIRS scanning safety eligibility test. The study was approved by the Korea University Anam Hospital institutional review board (IRB no. 2018AN0377). The study was conducted in accordance with the Declaration of Helsinki ethical principles.

### VR Therapy for SAD

**Figure 1:**
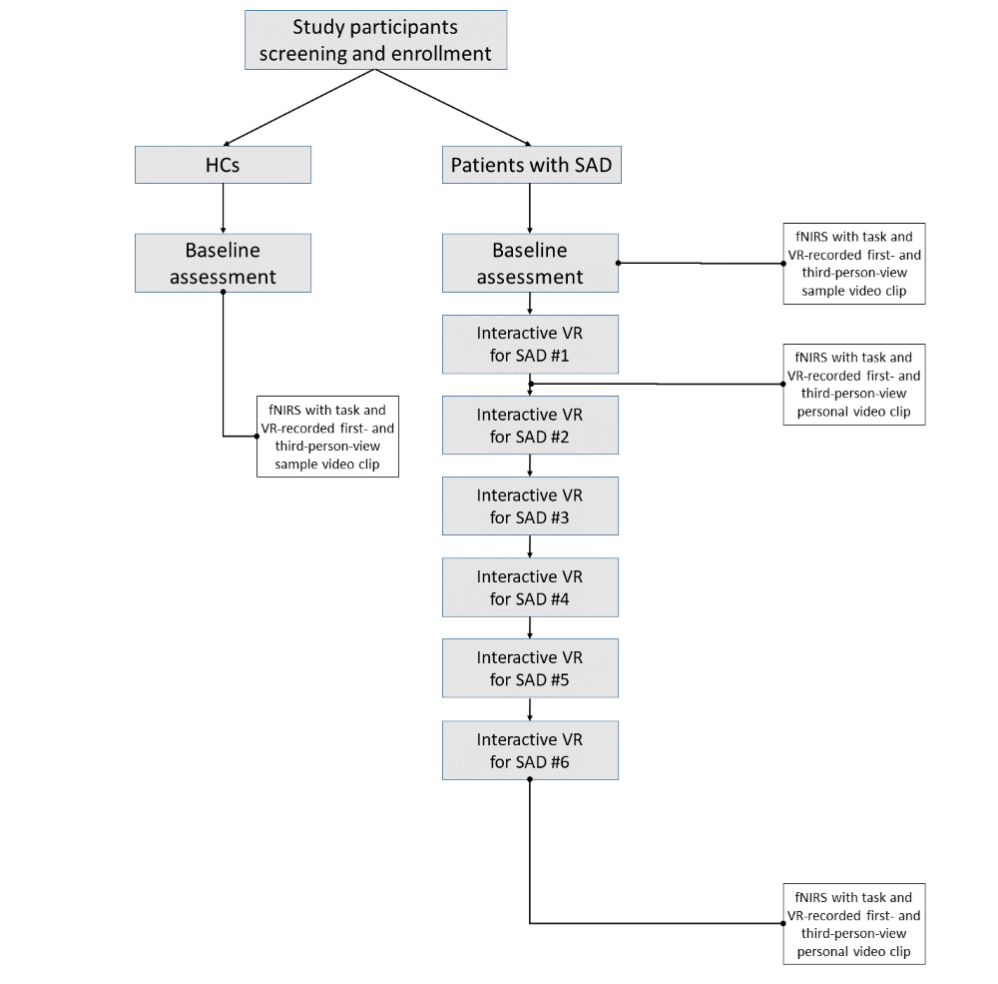
Overall schematic structure of the study. Patients with SAD and HCs were enrolled in a participatory and interactive VR treatment study. The HC group implemented fNIRS once by watching first- and third-person-view video clips at baseline. The SAD group implemented fNIRS 3 times in total at baseline, after the first and sixth sessions of VR treatment. fNIRS: functional near-infrared spectroscopy; HC: healthy control; SAD: social anxiety disorder; VR: virtual reality.

An overview of the participatory VR treatment for SAD is available in previous reports [29,33]. Here, a similar participatory VR program was used for the treatment of patients with SAD. The VR program had an introduction, a core, and a finishing phase, with 3 levels depending on the difficulty of the core phase. In the introductory phase, participants learned how to use VR for relaxation and meditation. During the core phase, virtual situations were set up. In these virtual setups, several participants met to perform team tasks. Participants were exposed to social situations and introduced themselves to an audience. The level of difficulty was gradually increased, and participants were required to engage in discussions, and the activities were repeated. Each video clip was recorded in first- and third-person views [34]. The mirroring techniques in treatment were modified, and a hybrid treatment was used in the study. Namely, we attempted to maximize the effectiveness of treatment by consulting or discussing with participants by reconfirming VR treatment performed by themselves from the first- and third-person views. Each participant underwent 6 sessions of the treatment program. Participants were allowed to select the difficulty level of the program according to their comfort level after the first session. The researchers supervised each session to ensure the safety of the participants. The psychological scales were evaluated before the commencement of the VR program and after its completion. When the participants were watching the video clip in first and third person, we measured neuronal activities in the desired brain regions using fNIRS before VR treatment initialization and after the first and sixth sessions (Figure 1).

### Clinical Symptom Assessments

The clinical symptoms of the participants were assessed using psychological scales. Subjects with SAD were scanned in 2 testing sessions, before and after VR therapy. We reported an analysis of psychological scale changes to evaluate the effectiveness of VR therapy for patients with SAD [29]. In this study, we studied the correlation between fNIRS results and psychological scales. Diagnostic assessment was measured using MINI [31]. Additionally, for social anxiety, we considered the Korean version of the Beck Anxiety Inventory (BAI) [35], and State-Trait Anxiety Inventory-X (STAI-X) [36]. Moreover, the Internalized Shame Scale (ISS) [37], the Post-Event Rumination Scale (PERS) [38], the Korean version of the Social Phobia Scale (K-SPS) [39], and the Korean version of the Social Interaction Anxiety Scale (K-SIAS) [39] were evaluated. Next, we also incorporated the Brief-Fear of Negative Evaluation (BFNE) scale [40], the K-SAD scale [41], and the Liebowitz Social Anxiety Scale (LSAS) [42].

For the HC group, fNIRS was evaluated once, whereas for the SAD group, it was measured 3 times (at baseline, after the first VR session, and after the sixth VR session). In each measurement, a 2-back task was conducted between the first- and third-person versions. We used a procedure similar to Wagner’s task [43]. Working memory in the SAD group was studied previously using the N-back task, in which the SAD group exhibited lower accuracy and longer response times than the HC group [44]. Similarly, cognitive function was assessed using the N-back task, which measured working memory. To provide human-recorded first- and third-person views, fNIRS was performed before and after the N-back operation to predict the expected therapeutic effect of VR-based psychotherapy. The 2-back task required the participants to determine whether the current stimulus was similar to the stimulus presented earlier in the trials. The task paradigm was composed of a block design that was repeated 3 times with alternate rest and task periods of 30 seconds.

### fNIRS Data Analysis

The fNIRS system (NIRSIT, OBELAB Inc., Seoul, Republic of Korea), which monitors activation in the PFC, is a portable, wireless, wearable, and multichannel (48 channels) brain imaging system that covers the entire forehead. Wavelengths of 780 and 850 nm were used, and 48 regions of light source and detectors separated by 3 cm were considered. The weight of the device is 550 g, and the sampling rate is 8.138 Hz. To remove high-frequency noise due to environmental artifacts and low-frequency noise due to the blood circulation in the body, the fNIRS signal was filtered using a bandpass filter with a cutoff frequency of 0.005-0.1 Hz. Considering the signal-to-noise ratio, the signal quality of each channel was evaluated after filtering, and values less than 30 dB were eliminated as unreliable.

Using the wavelength-dependent hemoglobin absorption coefficient and differential path length coefficient, the modified Beer-Lambert law was applied to the filtered signal, and changes in oxyhemoglobin (ΔHbO_2_) [45] and deoxyhemoglobin (ΔHbR) concentrations were extracted [46]. To account for the time-dependent change in hemodynamics, baseline correction was performed by subtracting the average from the calculated change in the oxygen concentration. Next, based on the Montreal Institute of Neurology standardization space (ICBM152), all 48 channels were grouped into 8 regions. After grouping each region of the PFC, the rejected channels identified during the channel rejection process were filled with the average ΔHbO_2_ of the channels allowed within each region.

### Statistical Analysis

The mean and standard deviation of demographic data, such as age and educational year, were calculated for both groups. After evaluating the variance equality through the Levene test, the difference in ΔHbO_2_ between the HC and SAD groups was analyzed by conducting an independent t test. One-way repeated ANOVA and post hoc analysis were conducted to understand subject variation in the SAD group from baseline. After the first and sixth VR sessions, the Box test for equivalence check and the Mauchly test of sphericity were conducted to analyze fNIRS data. Data processing was performed using the NIRSIT package in MATLAB (version 2019b; MathWorks Inc., Natick, MA, USA). Finally, we used the Pearson correlation coefficient to identify the relationship between psychological scale scores and area-specific changes in brain activation. Additional statistical analyses were performed using IBM SPSS Statistics 21 (SPSS Inc., Chicago, IL, USA), where the criterion for statistical significance was set at *P*<.05.

## Results

### Demographic Data

In this study, we analyzed the data from 28 patients with SAD and 27 people in the HC group. The mean age (SD) for patients with SAD were 23.74 (3.55) years, and for the HC group, they were 23.18 (3.27) years. The mean (SD) educational years for the SAD and HC groups were 15.00 (1.88) and 14.63 (1.37), respectively. No difference was observed between the groups in terms of age and years of education.

The mean (SD) K-SAD scores were 106.68 (15.33) for the SAD group and 48.93 (17.10) for the HC group. Significant differences were observed in the severity of SAD symptoms (Table 1).

**Table 1:** Demographic and clinical data between SAD^a^ patients and HCs^b^.

Demographic and clinical data	Patients with SAD	HCs
Gender, n (%) male/n (%) female	28 (9 [32%]/19 [68%])	27 (14 [52%]/13 [48%])
Age in years, mean (SD)	23.74 (3.55)	23.18 (3.27)
Education years, mean (SD)	15.00 (1.88)	14.63 (1.37)
K-SAD^c^ score^d^, mean (SD)	106.68 (15.33)	48.93 (17.10)
STAI-S^e^ score^d^, mean (SD)	49.21 (10.17)	35.48 (7.81)
STAI-T^f^ score^d^, mean (SD)	53.21 (10.30)	34.19 (7.19)
ISS^g^ score^d^, mean (SD)	51.86 (16.70)	15.22 (9.74)
BFNE^h^ score^d^, mean (SD)	44.39 (8.91)	27.74 (7.35)
PERS^i^ score^d^, mean (SD)	46.96 (8.34)	32.81 (11.61)
LSASanx^j^ score^d^, mean (SD)	38.86 (12.66)	13.96 (10.56)

^a^SAD: social anxiety disorder.

^b^HC: healthy control.

^c^K-SAD: Korean Social Avoidance and Distress.

^d^*P*<.01.

^e^STAI-S: State-Trait Anxiety Inventory-State.

^f^STAI-T: State-Trait Anxiety Inventory-Trait.

^g^ISS: Internalized Shame Scale.

^h^BFNE: Brief-Fear of Negative Evaluation.

^i^PERS: Post-Event Rumination Scale.

^j^LSASanx: Liebowitz Social Anxiety Scale-anxiety.

### Psychological Scale Scores of SAD and HC Groups

In this study, the scores of the SAD group were considerably higher than those of the HC group for all the tested scales that assessed associated anxiety symptoms (Table 1). In the SAD group, after VR treatment, the scores on the psychological scales for the SAD group decreased in all cases except 2, namely STAI-S (subscale of STAI, *P*=.11) and the Liebowitz Social Anxiety Scale-anxiety (LSASanx, subscale of LSAS, *P*=.16). (In a previous paper, we detailed the psychological scales used in the study and their purposes of assessment. Furthermore, we previously reported the changes in psychological scales according to VR treatment [29]).

### Baseline PFC Activity in SAD and HC Group Measure Using fNIRS

**Figure 2:**
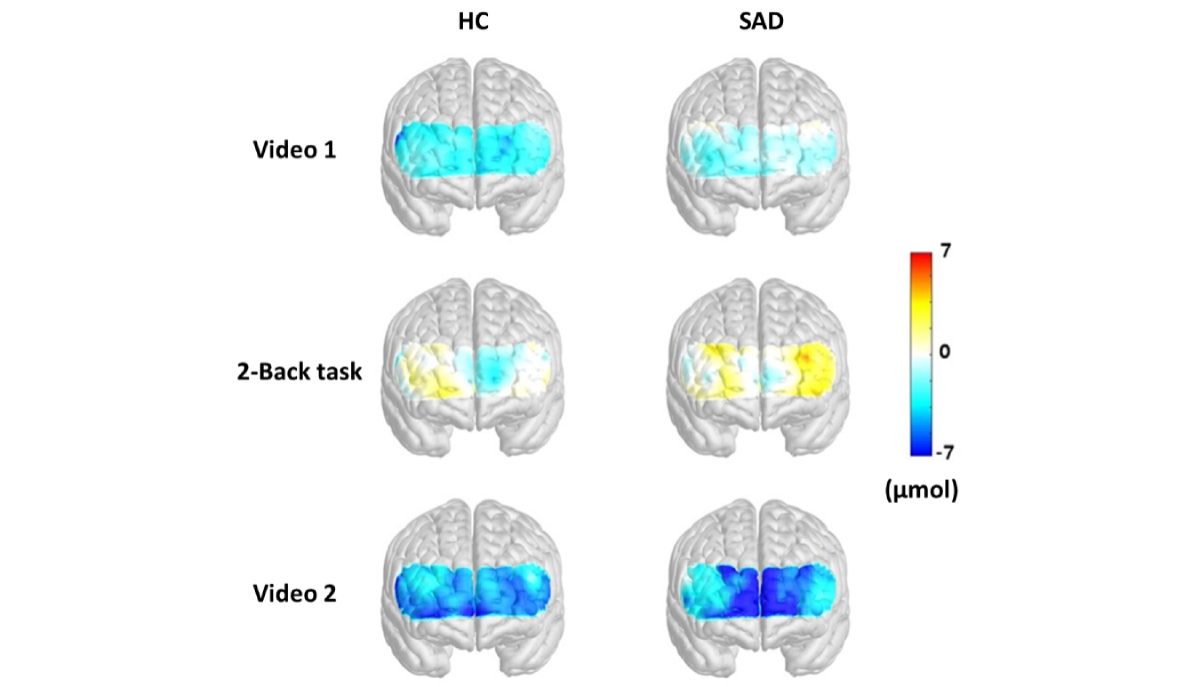
Activity of the PFC in the HC and SAD groups measured by fNIRS at baseline. When fNIRS was performed while both groups watched video 1, the activity of the right FPPFC was more decreased in the SAD group than in the HC group (*P*<.05). However, when implementing the 2-back task and watching video 2, there was no significant difference in the activity of the PFC between both groups. fNIRS: functional near-infrared spectroscopy; FPPFC: frontopolar prefrontal cortex; HC: healthy control; PFC: prefrontal cortex; SAD: social anxiety disorder.

We conducted fNIRS measurements for study participants before VR treatment to analyze brain activity in each region of the PFC, such as the dorsolateral prefrontal cortex (DLPFC), the VLPFC, the frontopolar prefrontal cortex (FPPFC), and the orbitofrontal cortex (OFC); see Figure 2. Brain activities among the SAD and HC groups were compared. A reduction in activation was measured by viewing first-person video clips (video 1), especially in the right FPPFC (*P*<.05). However, a negligible difference was observed between the two groups when the 2-back task was executed, and video clips in the third person were used for the treatment program (video 2). Next, while executing the 2-back task, activations in the left-brain areas of the HC group were lower than those of the SAD group, but the difference was not significant (Table 2).

**Table 2:** Comparison of the activation of the PFC^a^ measured by fNIRS^b^ between SAD^c^ patients and HCs^d^ at baseline.

PFC region		*t*		*df*		*P* value
**Video 1**						
	DLPFC^e^ (R^f^)	–1.8358	53		.07	
	VLPFC^g^ (R)	–0.9965	53		.32	
	FPPFC^h^ (R)	–2.0141	53		*.049* ^i^	
	OFC^j^ (R)	–0.4206	53		.68	
	DLPFC (L^k^)	–1.5172	53		.14	
	VLPFC (L)	–0.9989	53		.32	
	FPPFC (L)	–1.5577	53		.13	
	OFC (L)	–1.6796	53		.10	
**2-back task**						
	DLPFC (R)	–0.1724	53		.86	
	VLPFC (R)	0.3430	53		.73	
	FPPFC (R)	–0.5709	53		.57	
	OFC (R)	0.0677	53		.95	
	DLPFC (L)	–1.9085	53		.06	
	VLPFC (L)	–1.7692	53		.08	
	FPPFC (L)	–1.8762	53		.07	
	OFC (L)	–1.8244	53		.07	
**Video 2**						
	DLPFC (R)	0.2410	53		.81	
	VLPFC (R)	0.1600	53		.87	
	FPPFC (R)	0.6791	53		.50	
	OFC (R)	–0.3050	53		.76	
	DLPFC (L)	–0.1003	53		.92	
	VLPFC (L)	–0.2931	53		.20	
	FPPFC (L)	0.5063	53		.61	
	OFC (L)	–1.8244	53		.84	

^a^PFC: prefrontal cortex.

^b^fNIRS: functional near-infrared spectroscopy.

^c^SAD: social anxiety disorder.

^d^HC: healthy control.

^e^DLPFC: dorsolateral prefrontal cortex.

^f^R: right side.

^g^VLPFC: ventrolateral prefrontal cortex.

^h^FPPFC: frontopolar prefrontal cortex.

^i^*P* values in italics are significant (*P*<.05).

^j^OFC: orbitofrontal cortex.

^k^L: left side.

### Changes in PFC Activity in SAD After VR Treatment Using fNIRS

**Figure 3:**
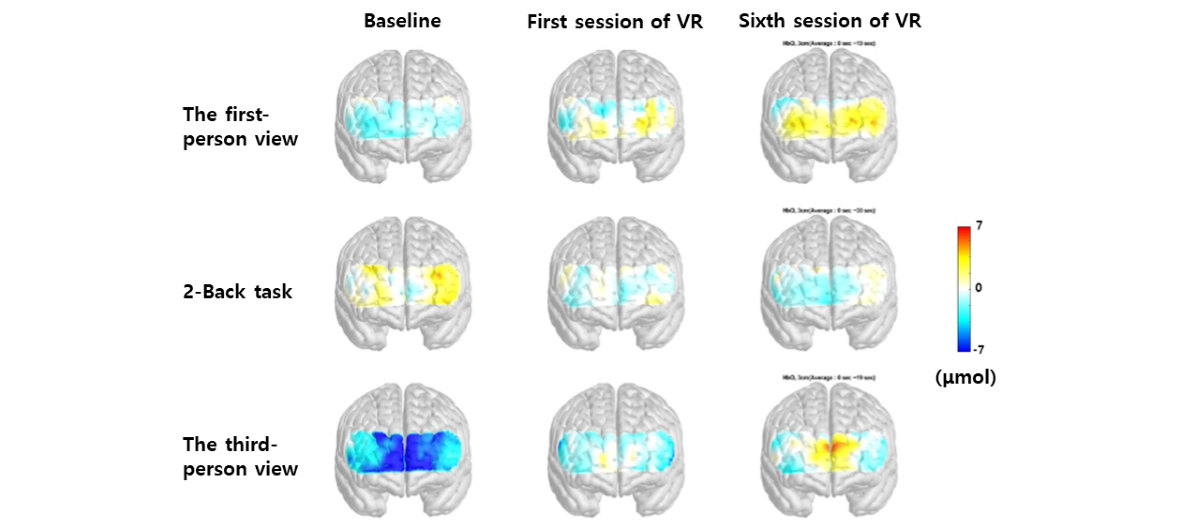
Activity of the PFC of patients with SAD at baseline, after the first VR session and after the sixth VR session. Comparing between baseline and after the first VR session, when watching video 2, there were significant differences in activity in the right FPPFC (*P*<0.01) and OFC (*P*<0.01) and in the left FPPFC (*P*<0.05) and OFC (*P*<0.05). Comparing between baseline and after the sixth VR session, when watching video 2, there were significant differences in activity in the right FPPFC (*P*<0.01) and OFC (*P*<0.05) and in the left FPPFC (*P*<0.05) and OFC (*P*<0.05). When patients with SAD were performing the 2-back task, there was a significant difference in activity in the right OFC (*P*<0.05) between baseline and after the sixth VR session. There was no significant difference in PFC activity when watching video 1. FPPFC: frontopolar prefrontal cortex; OFC: orbitofrontal cortex; PFC: prefrontal cortex; SAD: social anxiety disorder; VR: virtual reality.

We compared the brain activities measured by fNIRS (1) at baseline with those obtained after the first and sixth sessions of treatment in patients with SAD and (2) those obtained after the first session with those obtained after the sixth session. We observed significant differences in the right FPPFC (*P*=.01) and OFC (*P*=.045) and the left FPPFC (*P*=.03) and OFC (*P*=.04) when the treatment plan included video 2 for patients with SAD (Table 3 and Figure 3).

**Table 3:** Comparison of the activation of the PFC^a^ measured through fNIRS^b^ before and after VR^c^ treatment in SAD^d^ patients.

PFC region		*df*	Baseline vs session 1		Baseline vs session 6	
			*t*	*P* value	*t*	*P* value
**Video 1**						
	DLPFC^e^ (R^f^)	27	0.2855	.78	–0.3951	.70
	VLPFC^g^ (R)	27	–0.0279	.98	–0.6768	.50
	FPPFC^h^ (R)	27	–0.4071	.69	–1.2572	.22
	OFC^i^ (R)	27	–1.9413	.06	–1.5350	.14
	DLPFC (L^j^)	27	–0.7767	.44	–0.7083	.48
	VLPFC (L)	27	–1.8585	.07	–1.5250	.14
	FPPFC (L)	27	–0.8758	.39	–0.9972	.33
	OFC (L)	27	–0.1165	.91	–0.3891	.70
**2-back task**						
	DLPFC (R)	27	1.1630	.25	0.5698	.57
	VLPFC (R)	27	1.7643	.09	1.0058	.32
	FPPFC (R)	27	0.9091	.37	1.1983	.24
	OFC (R)	27	1.7939	.08	2.4581	*.02* ^k^
	DLPFC (L)	27	0.2466	.81	0.3295	.74
	VLPFC (L)	27	0.9830	.33	0.4685	.64
	FPPFC (L)	27	0.3272	.75	0.7516	.46
	OFC (L)	27	1.7245	.10	2.0201	.05
**Video 2**						
	DLPFC (R)	27	–1.7973	.08	–1.7163	.10
	VLPFC (R)	27	–1.2856	.21	–1.3256	.20
	FPPFC (R)	27	–3.1821	*.004* ^l^	–2.9260	*.007* ^l^
	OFC (R)	27	–2.8014	*.009* ^l^	–2.0984	*.045* ^k^
	DLPFC (L)	27	–1.8807	.07	–1.8464	.08
	VLPFC (L)	27	–0.9249	.36	–1.4879	.15
	FPPFC (L)	27	–2.3205	*.028* ^k^	–2.2514	*.03* ^k^
	OFC (L)	27	–2.6050	*.015* ^k^	–2.2136	*.035* ^k^

^a^PFC: prefrontal cortex.

^b^fNIRS: functional near-infrared spectroscopy.

^c^VR: virtual reality.

^d^SAD: social anxiety disorder.

^e^DLPFC: dorsolateral prefrontal cortex.

^f^R: right side.

^g^VLPFC: ventrolateral prefrontal cortex.

^h^FPPFC: frontopolar prefrontal cortex.

^i^OFC: orbitofrontal cortex.

^j^L: left side.

^k^*P* values in italics are significant (*P*<.05).

^l^*P* values in italics are significant (*P*<.01).

### Correlations Between Brain Activities and Psychological Scale Results

We analyzed the correlations between psychological scales and brain activities through fNIRS before and after VR treatment in patients with SAD. The STAI-S scores showed significant correlations with the activities of the right and left FPPFC, the right and left OFC, and the left DLPFC. Furthermore, the STAI-T scores were significantly correlated with the right and left OFC, and the ISS was significantly correlated with the right and left FPPFC and the right and left OFC in video 1. We also determined that STAI-S scores were significantly correlated with the right and left VLPFC, the right FPPFC, and the left DLPFC; the ISS was significantly correlated with the right and left DLPFC, the left VLPFC, the right and left FPPFC, and the right and left OFC; and the BFNE was significantly correlated with the right VLPFC in video 2. IN addition, PERS was significantly correlated with the right OFC, the K-SAD scale was significantly correlated with the left OFC, and LSASanx was significantly correlated with the right DLPFC, VLPFC, FPPFC and the right and left OFC while executing the 2-back task (Table 4).

**Table 4:** Correlations between brain activities and results of psychological scales.

Psychological scale			DLPFC^a^ (R^b^)		VLPFC^c^ (R)	FPPFC^d^ (R)		OFC^e^ (R)		DLPFC (L^f^)		VLPFC (L)		FPPFC (L)		OFC (L)
**Video 1**																
	BAI^g^	–.162		–.103		–.161	–.149		–.191		–.153		–.120		–.082	
	STAI-S^h^	–.321		–.199		–*.550*^i^	–*.527*^i^		–*.436*^j^		–.304		–*.466*^j^		–*.483*^i^	
	STAI-T^k^	–.109		–.097		–.329	*-.414* ^j^		–.259		–.241		–.296		–*.393*^j^	
	ISS^l^	–.297		–.175		–*.388*^j^	–*.400*^j^		–.307		–.191		–*.399*^j^		–*.439*^j^	
	PERS^m^	.075		.086		.213	.075		–.032		–.038		.031		–.070	
	SPS^n^	–.060		.053		–.117	–.117		–.088		–.009		–.113		–.060	
	SIAS^o^	.152		.127		.092	.090		.144		.083		.131		.132	
	BFNE^p^	.221		.208		.188	.350		.272		.135		.226		.269	
	K-SAD^q^	.086		.098		–.023	.246		.132		.187		.116		.227	
	LSASanx^r^	–.172		–.221		–.179	–.028		–.159		–.133		–.191		–.141	
	LSASavo^s^	–.073		–.097		–.021	.022		–.035		–.008		–.015		–.059	
**2-back task**																
	BAI	.172		–.035		.067	.003		.099		.014		.097		.122	
	STAI-S	.192		–.082		.144	.089		.044		–.182		.168		.065	
	STAI-T	.170		–.039		.153	.090		.078		–.022		.112		.052	
	ISS	.032		.164		.258	.037		–.014		–.096		.312		–.027	
	PERS	–.355		–.322		–.274	–*.426*^j^		–.091		–.106		–.318		–.187	
	SPS	–.155		–.360		–.028	–.269		.082		–.136		.026		–.280	
	SIAS	–.095		–.312		–.237	–.375		–.014		–.107		–.252		–.340	
	BFNE	–.102		.054		–.003	–.184		–.171		–.161		.103		–.255	
	K-SAD	–.027		–.182		–.178	–.230		–.009		–.133		–.115		–*.377*^j^	
	LSASanx	–*.457*^j^		–*.375*^j^		–*.395*^j^	–*.443*^j^		–.230		–.232		–.338		–*.474*^j^	
	LSASavo	–.312		–.240		–.303	–.335		–.252		–.192		–.284		–.369	
**Video 2**																
	BAI	.115		.096		.099	.146		.051		.071		.074		.118	
	STAI-S	–.359		–*.402*^j^		–.382^j^	–.234		–*.482*^i^		–*.460*^j^		–.325		–.357	
	STAI-T	–.091		–.157		–.147	–.035		–.257		–.230		–.136		–.181	
	ISS	–*.413*^j^		–.357		–*.426*^j^	–*.403*^j^		–*.452*^j^		–*.381*^j^		–*.432*^j^		–*.381*^j^	
	PERS	–.013		–.040		.081	–.002		.036		.062		–.052		.039	
	SPS	.041		.027		–.002	.020		.048		.007		.089		.168	
	SIAS	–.015		–.091		–.114	–.062		.068		.103		.060		.174	
	BFNE	.363		*.484* ^i^		.183	.175		.347		.370		.208		.234	
	K-SAD	.057		.013		–.148	–.072		.039		.072		–.028		.011	
	LSASanx	–.062		–.127		–.162	–.038		–.149		–.096		–.109		–.040	
	LSASavo	.045		–.007		–.059	–.009		–.018		.044		–.047		.024	

^a^DLPFC: dorsolateral prefrontal cortex.

^b^R: right side.

^c^VLPFC: ventrolateral prefrontal cortex.

^d^FPPFC: frontopolar prefrontal cortex.

^e^OFC: orbitofrontal cortex.

^f^L: left side.

^g^BAI: Beck Anxiety Inventory.

^h^STAI-S: State-Trait Anxiety Inventory-State.

^i^*P* values in italics are significant (*P*<.01).

^j^*P* values in italics are significant (*P*<.05).

^k^STAI-T: State-Trait Anxiety Inventory-Trait.

^l^ISS: Internalized Shame Scale.

^m^PERS: Post-Event Rumination Scale.

^n^SPS: Social Phobia Scale.

^o^SIAS: Social Interaction Anxiety Scale.

^p^BFNE: Brief-Fear of Negative Evaluation.

^q^K-SAD: Korean Social Avoidance and Distress.

^r^LSASanx: Liebowitz Social Anxiety Scale-anxiety.

^s^LSASavo: Liebowitz Social Anxiety Scale-avoidance.

## Discussion

### Principal Findings

To the best of our knowledge, this study is the first to determine the effects of VR therapy on SAD by using fNIRS measurements. From this study, we found that changes in activity in specific brain regions, such as the FPPFC and OFC, are closely related to VR treatment. In addition, we developed a VR program to enhance the efficacy of the treatment by creating first- and third-person views of exposure situations. In the first-person view, the patients directly present content to an audience, and in the third-person view, the patients visualize themselves speaking to an audience objectively.

The effects of VR treatment on patients with SAD were studied by measuring the changes in PFC brain activity using fNIRS. We were able to find that the activation of the right FPPFC was higher in the SAD group than in the HC group when watching video 1. This can be understood in a similar context as the blood flow of the MPFC decreases in situations in which anxiety is predicted in healthy individuals [47,48]. A positron emission tomography (PET) study reported that the blood flow in the right DLPFC, left inferior temporal cortex, and left amygdaloid-hippocampal region increases in patients with SAD [48]. However, using fNIRS analysis, we did not observe any difference in activation between the right DLPFC among the study groups. Previously, an fNIRS scan revealed that the activation change in the VLPFC in patients with SAD was less than that in the HC group during a verbal fluency task [49]. Here, the activation change in most regions of the left PFC was low and not significant.

In this study, we observed changes in the prefrontal area before and after VR treatment. Thus, activation in the right FPPFC and OFC and the left FPPFC and OFC significantly increased when patients with SAD were watching the video in the third-person view. By using PET after pharmacological treatments, Evans et al [50] reported that the regional brain metabolism of the ventromedial prefrontal cortex (VMPFC) in patients with SAD increases. Further, Hiser et al [51] reported that the activation of the anterior VMPFC increases upon pharmacological treatment or psychotherapy. We obtained similar results to these previous studies. However, the metabolism in the medial dorsal PFC decreased in patients with SAD after pharmacological treatment [52].

We obtained positive self-referential stimuli (positive words), neutral self-referential stimuli, and negative self-referential stimuli (negative words) after VR treatment to identify the activation changes in the brain region measured through fMRI in a previous study [33]. Therefore, patients with SAD exhibited increased activation in the right PCC/precuneus, lingual gyrus, left inferior temporal gyrus, precentral gyrus, and postcentral gyrus for positive self-referential stimuli. Patients with SAD also revealed increased activation in the left middle occipital gyrus, parahippocampus, left Rolandic operculum, and left caudate nucleus for negative self-referential stimuli. In this study, we observed the brain activity of participants watching the video clips, unlike the previous study, and determined the neural correlates by fNIRS, which were used to measure the activation of the prefrontal area, unlike fMRI, which can be used to measure the activation of the whole brain area.

Lastly, we identified correlations between psychological states in patients with SAD with brain areas activated upon VR therapy. The STAI-T scores and OFC activation, and ISS scores and prefrontal region activation (except the right VLPFC) were correlated. Studies have reported the correlation between the severity of SAD symptoms and the activation of certain brain areas. Klumpp et al [53] reported that improvements in the symptoms are correlated with the increase in the activation of brain areas, such as the medial orbitofrontal and dorsomedial frontal gyrus. Marin et al [54] reported that hypoactivation of the VMPFC is correlated with the severity of anxiety. Based on fNIRS analysis, Yokoyama et al [49] reported that changes in VLPFC activation are negatively correlated with social fear and activation of the left DLPFC is positively correlated with social anxiety [55].

Studies have reported that amygdala activation is high in patients with SAD [48,56]. Here, we could not study amygdala activation, because measuring subcortical brain regions with fNIRS is difficult. The VMPFC and the VLPFC are known to be associated with the amygdala in patients with SAD. Generally, the VMPFC regulates social functions and inhibits the amygdala to regulate the fear response [51]. Thus, increased activation of the ventromedial prefrontal regions from social anxiety treatments is beneficial. In addition, the degree of functional connectivity between the VLPFC and the amygdala is negatively correlated with the severity of anxiety [57]. This result supports our results that activation of the VLPFC and the degree of anxiety treatment are correlated.

The blood flow in the right DLPFC decreased in patients with SAD during the repetition task [48]. We observed that activation of the right OFC during the 2-back task in our study increased after treatment, which contradicts the results of some previous studies. Further, regional cerebral blood flow of the rhinal cortex, amygdala, hippocampal region, and parahippocampal region decreases in patients with SAD during task performance. The regional cerebral blood flow of the middle frontal cortex and dorsal ACC changes in patients with SAD, particularly in patients who effectively perform the task [58]. We obtained similar results in our study.

Differences were observed in brain activation in high anxiety–provoking situations rather than in low anxiety–provoking situations [59]. Patients with SAD were more anxious during the first-person view than during the third-person view, which resulted in differences in activation in the HC group. In addition, the changes in brain activation appeared earlier when the video in the third-person view was used, because the patients felt less anxious. However, the effects of the treatment appeared later when the video in the first-person view was used, probably because the patients felt more anxious. Therefore, the effectiveness of the treatment modality should be verified by conducting more treatment sessions.

### Limitations

This study had some limitations. First, the sample size was relatively small. However, our analysis was thorough, and we were able to investigate functional brain activity after VR therapy in patients with SAD. Second, the number of sessions for VR treatment was small, and the follow-up period was relatively short. We observed the changes in psychological scale scores and brain activities measured through fNIRS after VR treatment. However, after the sixth session of treatment, the anxiety levels observed on most psychological scales in the SAD group were higher than in the HC group. This could be because changes in brain activity due to VR treatment were not prominent as yet. Perhaps more treatment sessions are required. Thus, more VR treatment sessions should be conducted for a larger sample size to observe prominent therapeutic benefits. Third, we could not measure the brain activity in real time. Applying electroencephalography to a VR head-mounted display (HMD) has enabled measurement of brain waves [60]. However, we were not able to apply fNIRS and VR HMD because of several technical limitations. Lastly, we could not observe changes in brain areas other than the PFC (especially amygdala), owing to mechanical limitations of portable fNIRS. Although fMRI has the advantage of being able to perform comprehensive and in-depth measurement of the entire brain function, it is difficult to apply various tasks and has a high economic burden, so there are obvious limitations in actual clinical application. However, in terms of cost and usability, portal fNIRS has a high possibility of being applied to actual clinical practice in combination with VR, so it is worthy of overcoming the disadvantages of measurement compared to fMRI.

### Conclusion

We conducted VR therapy on patients with SAD to prove the treatment effects after developing a VR treatment program that was exposed to social situations in the first- and third-person views. We identified the brain activity changes in the FPPFC and OFC in patients with SAD after VR treatment in this study, which can provide functional clues in specific brain area for VR treatment. In addition, we found that the STAI-T scores were correlated with the activity of the OFC and the ISS scores were correlated with most of the PFC except the right VLPFC. This can be a clue that the change after treatment in some psychological domains in SAD is related to a specific PFC region. Future studies are required to identify and confirm the neural correlates of VR treatment in patients with SAD for sufficient periods in large sample sizes.

## Abbreviations

ACC: anterior cingulate cortex
BAI: Beck Anxiety Inventory
BFNE: Brief-Fear of Negative Evaluation
DLPFC: dorsolateral prefrontal cortex
DSM-5: Diagnostic and Statistical Manual of Mental Disorders, Fifth Edition
fMRI: functional magnetic resonance imaging
fNIRS: functional near-infrared spectroscopy
FPPFC: frontopolar prefrontal cortex
HbO_2_: oxygenated hemoglobin
HbR: deoxygenated hemoglobin
HC: healthy control
HMD: head-mounted display
ISS: Internalized Shame Scale
K-SAD: Korean Social Avoidance and Distress
K-SIAS: Korean version of the Social Interaction Anxiety Scale
K-SPS: Korean version of the Social Phobia Scale
LSAS: Liebowitz Social Anxiety Scale
MINI: Mini-International Neuropsychiatric Interview
MPFC: medial prefrontal cortex
OFC: orbitofrontal cortex
PCC: posterior cingulate cortex
PERS: Post-Event Rumination Scale
PET: positron emission tomography
PFC: prefrontal cortex
SAD: social anxiety disorder
STAI: State-Trait Anxiety Inventory
VLPFC: ventrolateral prefrontal cortex
VR: virtual reality
